# Catatonic syndrome associated with lead intoxication: a case report

**DOI:** 10.4076/1757-1626-2-8722

**Published:** 2009-08-11

**Authors:** Mohammad Jafar Modabbernia, Ali Reza Mirsafa, Amirhossein Modabbernia, Farhad Pilehroodi, Maryam Shirazi

**Affiliations:** 1 Department of Psychiatry, Guilan University of Medical Sciences, Iran; 2 Shafa Hospital15 Khordad Ave, RashtIran 41939-55599

## Abstract

**Introduction:**

Little is known about catatonia associated with lead intoxication.

**Case presentation:**

A retired printing house worker man presented with one week history of refusal to eat and mutism. He was treated with possible diagnosis of catatonia with administration of Lorazepam 3 mg P.O. daily. Significant improvement occurred after 48 hours. In further examinations, there was no evidence of physical and mental disorders while impairment in neuropsychiatry test, identification of Dohle body, basophilic stippling and toxic granulation in peripheral blood smear and blood lead level of 12.8 μg/dl were recorded.

**Conclusion:**

Possibly, lead intoxication results in changes in neurotransmitter system that leads to catatonia. Lorazepam improves patient’s condition through changes in this system.

## Introduction

Catatonia is a clinical syndrome with main features such as motor immobility and waxy flexibility. Mutism, extreme negativism and echolalia may also present as a part of its clinical manifestation.

Catatonia is associated with psychiatric conditions (schizophrenia, mood Disorders), medical conditions (head trauma, brain tumors, infections like encephalitis, metabolic disorders), neurocognitive disorders, metabolic disturbances [1] and metal poisoning [2]. There is little known about epidemiology of psychiatric disorder associated with metal intoxication [3].

## Case presentation

A 45-year-old married Persian white male who was a retired printing house worker presented with refusal to eat and drink, mutism and lack of response to external stimuli and was taken to a psychiatric center by his family members. He was suffering from sleep disturbance and lack of appetite from two months earlier subsequent to self burning of one of his child. His other child was attending the military service and this is an additional stressful event for him .also as his family stated he had been indifferent to his life affairs for a long time. Besides he had a history of problem in defecation (constipation) and abdominal pain for several years. One week before hospital admission, he gradually became stuporous and mute. History of cigarette smoking, drug abuse and alcohol consumption was negative. He had a prior history of taking part in war, 23 years ago and in his family history, one of his child and his sister were under treatment because of mental retardation and mood disorder respectively. Patient’s weight and height were 65 kgs and 168 cm respectively. Regarding mentioned symptoms and signs, it was impossible to take a history from the patient and do physical examination until 48 hours after initiation of treatment.

**Figure 1.**
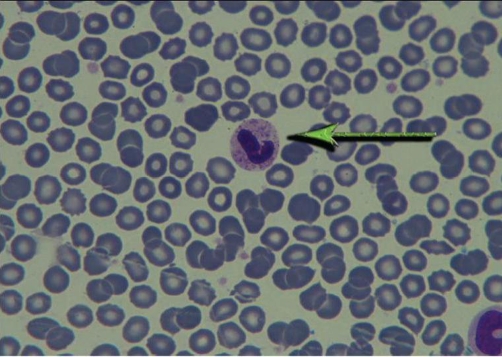
Toxic granulation.

**Figure 2.**
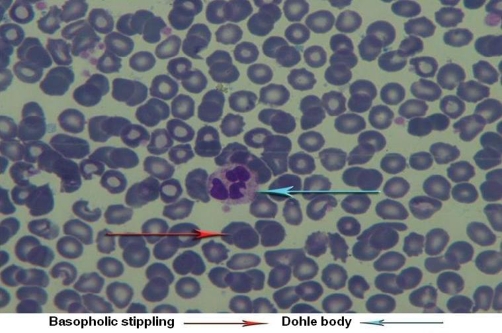
Dohle body, and basophilic stippling.

**Figure 3.**
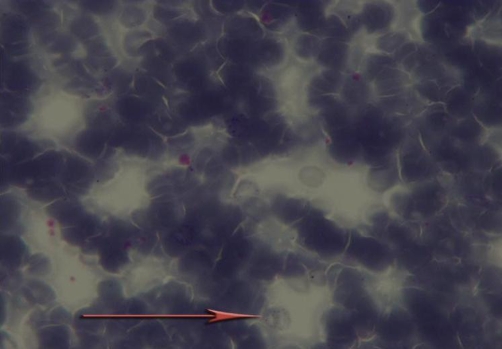
Basophilic stippling.

Therefore, with possible diagnosis of catatonia, Lorazepam [5] 3 mg through nasogastric tube was prescribed. 48 hours after receiving Lorazepam, a marked improvement in catatonic features was seen. His motor ability improved and he was able to give relevant answers to the questions. In clinical interview and physical examination, delusion, hallucination, major depression symptoms and signs and disturbance of consciousness were not detectable and schizophrenia, major depression and delirium as possible causes of catatonic syndrome were ruled out. Considering patient’s occupation (printing-house worker), complete physical examination was performed. After we did physical examination, it became clear that he has been suffering from fingers’ paraesthesia. Other parts of general physical examination including head and neck, heart and lungs, abdominal, extremities are all normal. in the neurologic examination MMSE score in registration, 3 stage command, reading, writing, and copying was abnormal (21 of 30) and go/no go test was impaired, other components on neurologic examination including cranial nerve, motor, sensory, cerebellar and gait (which is done carefully by a neurologist) were normal. In Para clinic study, neuropsychology test showed dysfunction in frontal lobes and in the study of PBS, toxic granulation (Figure 1), Dohle body (Figure 2), and basophilic stippling (Figure 3) were seen. (In order to detect basophilic stipplings more efficiently, RBCs were packed with micro hematocrit method and after Wright-Gimsa staining, these cells were seen more clearly).

These abnormal cells were diagnostic for inflammatory and infectious diseases, megaloblastic anemia and thalassemia [4] while in physical and laboratory examinations no evidence of these disorders was found. BLL was measured and it was 12.8 μg/dl. Routine laboratory examination including blood count, serum electrolytes and biochemistry, renal and thyroid function tests was normal.

Catatonic state resolved within 48 hours after beginning the treatment [5] and did not return after that. Patient have done well in follow up visits arranged every 2 months although cognitive dysfunction did not resolve completely and he was referred to a neurologist for treatment of his cognitive problems.

## Discussion

Catatonic syndrome may present as a clinical manifestation in schizophrenia, major depression with psychotic feature, delirium, brain disorders, hepatic encephalopathy, seizure disorders, drugs and rarely metal poisoning.

Reported patient presented with catatonic features and made a dramatic improvement 48 hours after receiving Lorazepam 3 mg P.O. schizophrenia, major depression and delirium in the absence of mental and physical manifestations of these conditions were nearly ruled out. Because of absence of history of drug abuse or intake, hepatic and renal dysfunction or electrolyte imbalance these conditions were also excluded. Besides because the problem of the patient began gradually, non convulsive status epilepticus was not considered a suitable diagnosis.

In addition to above points, association between catatonia and metal intoxication [3], cognitive impairment, patient’s occupation (printing-house worker),presence of paraesthesia and constipation, identification of abnormal cells in PBS (Dohle body, basophilic stippling and toxic granulation) and BLL of 12.8 μgr/dl ( because of unethical aspect of performing bone marrow Biopsy, there is no information about lead on bone marrow cells in this patient) catatonia associated with lead intoxication was the most possible diagnosis in this case.

Mechanism of catatonia is not fully understood yet. Many hypotheses about changes in different neurotransmitter systems has been emerged; one of the most important of them is a “top down” modulation of basal ganglia secondary to reduction of cortical GABA [6]. Other mechanisms includes blockade of dopaminergic system [7] as well as hyperactivity of glutamatergic system [8].On the other hand, One of the changes in chronic lead intoxication is elevation of glutamate and reduction of GABA levels [9,10] in the brain as well as reduction of dopaminergic activity in basal ganglia [11]. It seems that lead impact (through changes in interaction between dopaminergic and GABA-ergic and possibly glutamatergic system) in addition to family history of mood disorders and having a lead-related occupation (working in printing-house) act as predisposing factors which in an interaction with environmental stress (child self burning and second child departure) resulted in clinical manifestations of catatonia. Identification of abnormal cells in PBS, impaired neuropsychologic tests and quick response to Lorazepam (possibly through activation of GABAergic receptor and regulation of neurotransmitter system) bold the role of lead in catatonic syndrome.

## Conclusion

Lead impact in an interaction with other bio-psycho social factors was considered as the best explanation for the patient’s condition. Early diagnosis of this disorder may lead to 1. Preventing unnecessary treatments and long-term prescription of neuroleptic drugs. 2. Reduce patient and his/her family worry.

## Abbreviations

BLL: Blood lead level
PBS: Peripheral blood smear
